# Physiological Characteristics and Operational Performance of Pilots in the High Temperature and Humidity Fighter Cockpit Environments

**DOI:** 10.3390/s21175798

**Published:** 2021-08-28

**Authors:** Biyun Zhou, Li Ding, Bo Chen, Huijuan Shi, Yingfang Ao, Ruiqi Xu, Yan Li

**Affiliations:** 1 Beijing Advanced Innovation Center for Biomedical Engineering, School of Biological Science and Medical Engineering, Beihang University, No. 37 XueYuan Road, HaiDian District, Beijing 100191, China; biyunzhou@buaa.edu.cn (B.Z.); 07441@buaa.edu.cn (L.D.); suzumiya@buaa.edu.cn (B.C.); shihuijuan1103@buaa.edu.cn (H.S.); aoyingfang@163.com (Y.A.); richxu96@buaa.edu.cn (R.X.); 2 Institute of Sports Medicine, Peking University Third Hospital, Beijing Key Laboratory of Sports Injuries, Beijing 100191, China

**Keywords:** fighter cockpit, high temperature, combat capability, physiological characteristics

## Abstract

During military operations in high-temperature and relative humidity (RH) conditions, the physiological state and combat capability of pilots are affected severely. In a fighter cockpit, experiments were conducted on thirteen voluntary subjects wearing pilot suits at 21 °C/30%, 30 °C/45%, and 38 °C/60% RH, respectively, in order to examine the physiological changes of pilots in combat thoroughly. The target strike performance, core and skin temperatures, pulse rate, and other parameters were measured and investigated. Significant inter-condition differences were noted in the pulse rate, core temperature, mean skin temperatures, and sweat amount, which increased markedly with elevating temperature and RH. Contrastively, blood oxygen saturation (SpO2) dropped with such elevations. Concerning the skin temperature, the chest and back skin temperatures remained stable, while the temperatures at the hands, feet, and lower arms underwent larger changes with the increasing temperature and humidity. At 38 °C/60% RH, the sweat amount was 3.7 times that at 21 °C/30% RH. The subjects’ operational error rates increased as the core temperatures rose, showing high correlations (r^2^ = 0.81). The results could serve as a theoretical basis for the design of pilot protective equipment and the control of aircraft cockpit temperature.

## 1. Introduction

Researchers generally regard the living environment above 35 °C and the production environment above 32 °C as high-temperature environments and deem the environment with RH above 60% as a high-humidity environment [1]. In the aviation field, extreme flight conditions are often encountered. For example, a high-temperature and -humidity environment is common, where the maximum temperature can rise above 40 °C and the RH can reach over 60%. According to reports by Bollinger, the cockpit temperatures of the U.S. F-4 fighter jets and A-10 attackers could exceed 45 °C during low-altitude flights in a hot climate [2,3]. In the case of China, during the high-temperature season, the J11 fighter unit pilots at the airports in the south of the Yangtze River were affected by high temperatures and humidity during the pre-takeoff run-up, low-altitude flight, or first-degree combat readiness duty [4]. The bladder anti-G suit for fighter pilots also contributes to the heat stress [5], which is a very serious issue for the pilots, given the limited role of the refrigeration system inside the aircraft. [6]. This would become a prominent thermal physiological problem, especially in the stage of pre-flight [7]. To this end, this study explores the effects of high-temperature and -humidity environments on the physiological characteristics and combat performance of pilots during flight operations, which is profoundly meaningful for developing precise pilot protection measures, thereby ensuring flight safety.

Massey et al. found that the accident rates peaked in the summer months, and heat stress was an important problem [8], revealing a great impact of high temperature and humidity on the pilots’ operational performance. Studies have shown that high thermal loads in the cockpit are likely to cause thermal discomfort, dehydration, and fatigue as well [9,10]. In addition, working in a high-temperature environment may lead to heat stroke and heat cramps [11]. The effects of air temperature and RH on the human thermal response were explored by Hayakawa at various metabolic levels, which found an intensifying influence of RH on the human body with the rise in air temperature and metabolic level [12]. Overall, high humidity causes human discomfort from two aspects: the increase in air humidity leads to the increase in skin wetness [13], and the high humidity causes insufficient evaporative heat loss in turn, which results in discomfort of the human body [14,15]. An enclosed hot and humid environment can pose serious physical and psychological effects on the human body, causing symptoms such as physical weakness, confusion, and even fainting, which may result in operational errors [16,17].

In some predictive model-based studies in the thermal environment, the decline in attention was found apart from the physiological effects [18,19,20], which would also contribute to the risk of unsafe operating behaviors. According to a study by HILDE et al., wearing protective clothing in high-temperature environments might lead to an elevation of core temperature and the risk of pilot errors as well [21]. Nunneley et al. investigated human performance at rather high temperatures of 35 °C and 26 °C in a simulated aircraft cockpit environment and found similar conditions in the aircraft can be associated with impaired performance, particularly in emergency situations. Hong et al. studied soldiers’ cognition experiments under 22 °C and 35 °C and found that soldiers’ reaction times increased under high temperature [22]. When actual human body temperature increases, the performance of a vigilance task will noticeably alter in hot environmental conditions [23]. The above research was conducted in simulated cockpits, which differed tremendously from the real cockpits in terms of airtightness and space size. Thus, their findings may differ from those conducted in real combat scenarios.

To accurately grasp the physiological characteristics and operational performance of pilots in high temperature and humidity flight environments, experiments are carried out in this study by setting up simulation environments in the cockpit of a certain retired fighter aircraft. Changes in their physiological characteristics and operational performance are investigated during simulated combats under three environmental conditions. Furthermore, the correlations between physiological characteristics and operational performance are explored. Compared to previous experiments, the present study can conduct flight operations in the cockpit of a real aircraft, reflecting the physiological changes and the combat performance of pilots in combat conditions more precisely. This study could also provide a theoretical basis for the design of the pilot ventilation suit and the temperature adjustment of a fighter cockpit.

## 2. Materials and Methods

### 2.1. Subjects

In accordance with the basic standards on anthropometric parameters of pilots [24], thirteen healthy male college students were recruited as volunteers for the present simulation experiments of pilot thermal physiology. Given the fact that the majority of pilots are men, no female subjects were included. None of them had any history of heart disease, physical disabilities and other related diseases. Their average age was 23.6 years (23–25 years), average height was 170.9 cm (165–177 cm), and mean weight was 61.67 kg (53–70 kg). Before the experiment, the volunteers were informed of the purpose and procedure of the experiments, and research-related risks, and each of whom signed an informed consent form. The experiments were approved by the ethics committee. They also received simulated flight training 3 times at room temperature, with a duration of 4 h each time, and finally could all complete three missions of target striking within 15 min. The subjects did not experience vertigo in these pre-experiments.

### 2.2. Experimental Equipment

The equipment used in the present experiments included a 3rd-generation aircraft fighter cockpit, a flight simulator, a cockpit temperature and humidity control system, pilot clothing, a pulse oximeter, temperature sensors, an electronic scale, etc.

Pilot clothing: The subjects wore anti-G suits; the thermal resistance and the evaporative heat dissipation index were 1.79 clo and 0.41.

**Figure 1:**
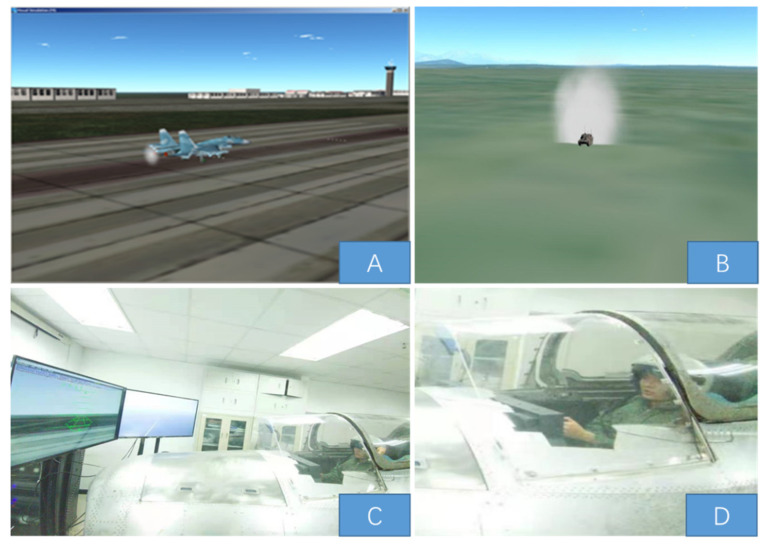
Fighter visual simulation (**A**), target visual simulation (**B**), the displays (**C**), fighter cockpit (**D**).

Flight simulator: As shown in Figure 1, the flight simulator comprised a visual system, a simulated cockpit, a console system, and an operating system. The cockpit includes an operating lever, instrument panel (Airspeed Indicator, Attitude Indicator, Altimeter Heading Indicator, etc.), ejection seat, temperature and humidity sensors, target lock and launch button, etc. The visual system generated scenes outside the cockpit via a computer imaging system, including the airport runways, buildings, fields, roads, and landforms. In this simulator, the pilots could perform operations in the ground target missions.

Cockpit environment control system: The experiment used the HFW (hot & humidity flow work) series, which is a precise system for controlling constant temperature and humidity in the cockpit environment. This system has controlling ranges over 10–50 °C and 20–90% RH, with the precision of temperature and humidity being ±0.2 °C and ±5%, respectively.

Pulse oximeter: The CMS50D+ (Contec Medical SpO2), with an SpO2 range of 0–100% and measuring accuracy of ± 2%, was used as the pulse oximeter in the experiments.

Temperature sensor: The skin and oral temperatures of subjects were measured with iButton temperature and humidity loggers from Dallas Semiconductor. The sampling rate was set at 1 time/min, with a measuring range of −40 to 80 °C and a measuring accuracy of 0.1 °C.

Precision scale: A Mettler Toledo precision scale was used to measure the sweat amount at an accuracy of ±1 g.

### 2.3. Experimental Protocol

The experiments were carried out within the fighter cockpit. In accordance with the Fighter Index of Thermal Stress (FITS) of United States Air Force [25], the cockpit temperature and humidity were set at three levels: comfortable environment, 21 ± 0.2 °C, 30 ± 5% RH; moderate environment, 30 ± 0.2 °C, 45 ± 5% RH; and intense environment, 38 ± 0.2 °C, 60 ± 5%RH. The subjects experienced the experimental conditions in assigned random orders. The experiments were conducted between 9:00 and 10:00 a.m. or 15:00 and 16:00 p.m., and each subject was experimented on at least 24 h after the previous experiment.

The experimental contents were identical under the three conditions, with each experiment containing three missions. After adjusting the cockpit ambient temperature, the subjects wore anti-G suits and entered the fighter cockpits with measuring sensors to perform the flight missions. The subjects were required to complete the target strike missions within 15 min. In the meantime, their physiological indicators were monitored, including pulse rate, SpO2, skin temperature, oral temperature, and so on. Prior to the experiments, three ground targets in “×, ■, ▲” shapes were set up, which represented different actual targets. At the beginning of the experiments, the subjects were asked to take off from the same position by controlling the flight simulator. When the aircraft climbed up to 3000 m, the subject began to search for targets with radar. If the subjects found the targets, they should hit the target after aiming carefully, and the simulation system recorded the number of target strikes. A flight mission was deemed successful if all the three ground targets were hit and no crash. If the mission was failed or the experiment time exceeded the averaged target striking time per target plus two standard deviations, the trial was considered as invalid. If the target striking task was completed ahead of schedule, the subjects still needed to continue flying with the same flight performance to reach the time length of 15 min. Upon completion of each flight, the subjects sat and rested in the cockpit for 5 min, and then filled out a subjective questionnaire on the seven-point ASHRAE scale, as shown in Table 1. This step was also repeated after the other two flight trials. The average total exposure time of participants under each environmental condition was 55 min.

### 2.4. Subject Parameter Measurement

**Figure 2:**
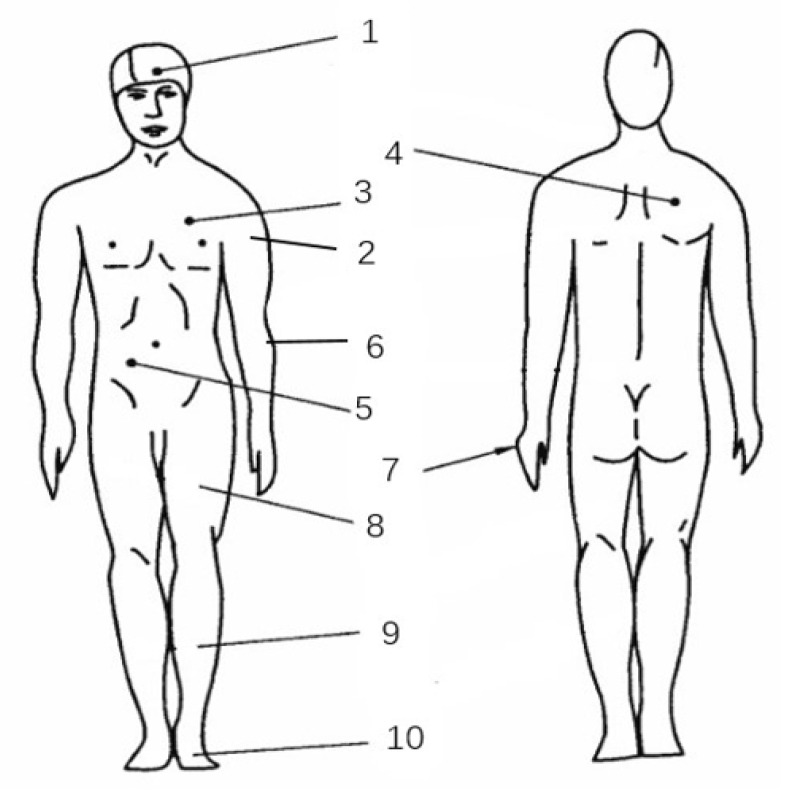
Measuring points of human skin temperature.

The measuring sites for human skin temperature are demonstrated in Figure 2, where points 1–3 represent the skin temperatures of the head, left upper arm, and chest. Further, sites 4–7 represent the back, abdomen, left lower arm, and left hand, respectively, and sites 8–10 represent the left thigh, left calf, and left foot.

The weighted mean skin temperature $T_{sk}$ was calculated by employing the 10-point weighted coefficient method [26]. The relevant computational formula is shown in Equation (1). The core temperature was calculated as the oral temperature plus 0.3 °C [27]. Although the body temperature may vary with age, weight, and gender, this calculation method is applicable to the skin temperature calculation of pilots.

(1) $$T_{sk}=0.06T_{1}+0.12T_{2}+0.12T_{5}+0.08T_{3}+0.12T_{4}+0.06T_{6}+0.05T_{7}+0.19T_{8}+0.13T_{9}+0.07T_{10}$$

The subjective thermal sensation was assessed using the seven-point scale proposed by ASHRAE. In this study, only the overall thermal sensation was surveyed.

### 2.5. Statistical Analysis

All statistical analyses were performed using SPSS 24.0 and Origin 2018 software at a significance level of α < 0.05. We Shapiro–Wilk tested all the data to verify normality. A two-way analysis of variance (ANOVA) with repeated measures was utilized to analyze how the physiological characteristics of subjects were affected by heat conditions (21, 30, 38 °C) and task repetition (task1, task2, task3). A Friedman test was also utilized to analyze the significance of the mean error rate.

The logarithmic function was used to fit the relationship between the human body temperature and time, as described by Equation (2):

(2) T=a−bln(t+c) 

where T and t stand for the human body temperature and time, respectively, and a, b, c are the relevant fitting coefficients.

## 3. Results 

### 3.1. Core Temperature

**Figure 3:**
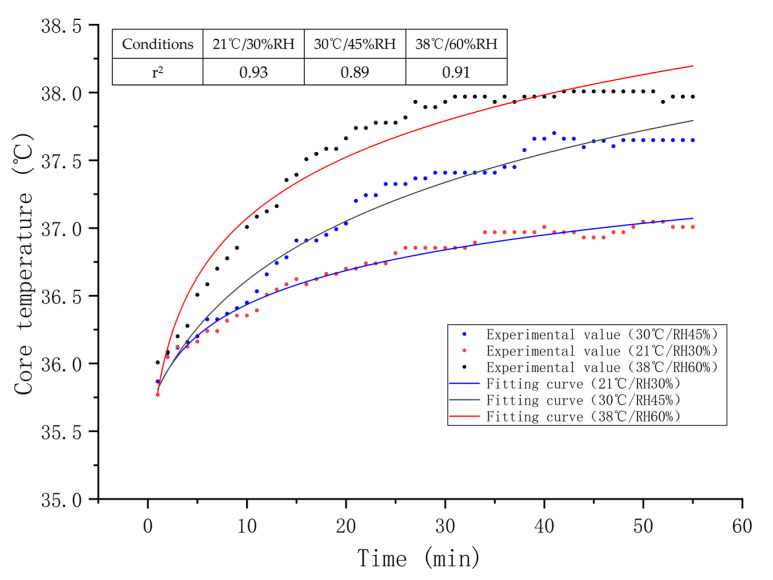
The core temperatures and logarithmic fitting curves versus time under three conditions, and high values of r^2^ indicate goodness of fit.

Figure 3 illustrates the temporal changes in the core temperature of subjects during the simulated flight under 21 °C/30%, 30 °C/45%, and 38 °C/60% RH, respectively. The core temperatures increased rapidly within 25 min after experiment initiation, and then tended to increase slowly. In this study, the logarithmic function was used to fit the relationship between core temperature and time, and the fitting degree of the model was good (*p* < 0.001). The definition of logarithmic function is shown in Equation (2). As can be seen from Figure 3, the core temperature increases with the working time in the cockpit environment logarithmically, while the standard deviation of core temperature decreased with time. At 38 °C/60% RH, the maximum core temperature reached 38.0 °C. Although the initial core temperatures were fundamentally identical, there were differences in core temperature among three different environments after 15 min (*p* < 0.001), and the heating condition and time had a significant interaction on the core temperature.

### 3.2. Skin Temperature

**Figure 4:**
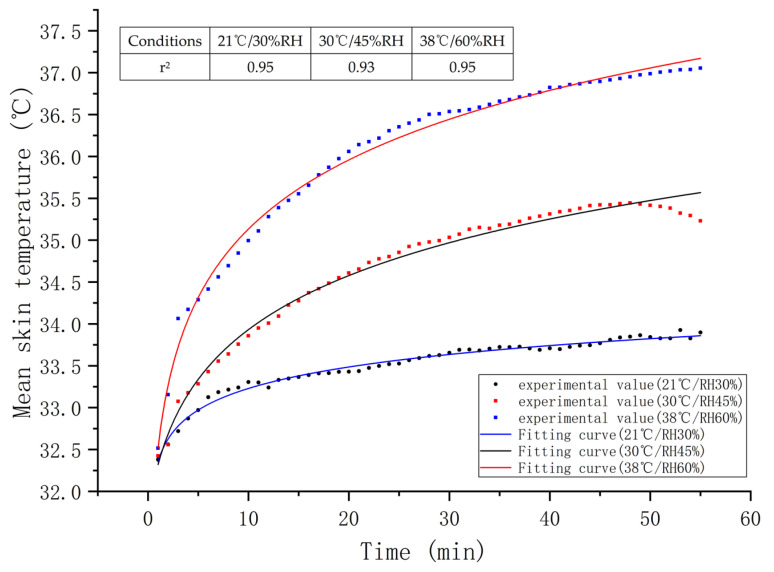
The weighted mean skin temperatures and logarithmic fitting curves versus time under three conditions, and high values of r^2^ indicate goodness of fit.

Figure 4 shows the changes in the weighted mean skin temperature of the subjects under three experimental conditions. In this study, the logarithmic function has been used to fit the relationship between mean temperature and time, and the function is shown in Equation (2). As shown in Figure 4, the fitting degree of the model was good (r^2^ > 0.9, *p* < 0.001). It could also be found that the weighted mean skin temperature of the human body grew along with the increase in the ambient temperature (*p* < 0.01).

**Figure 5:**
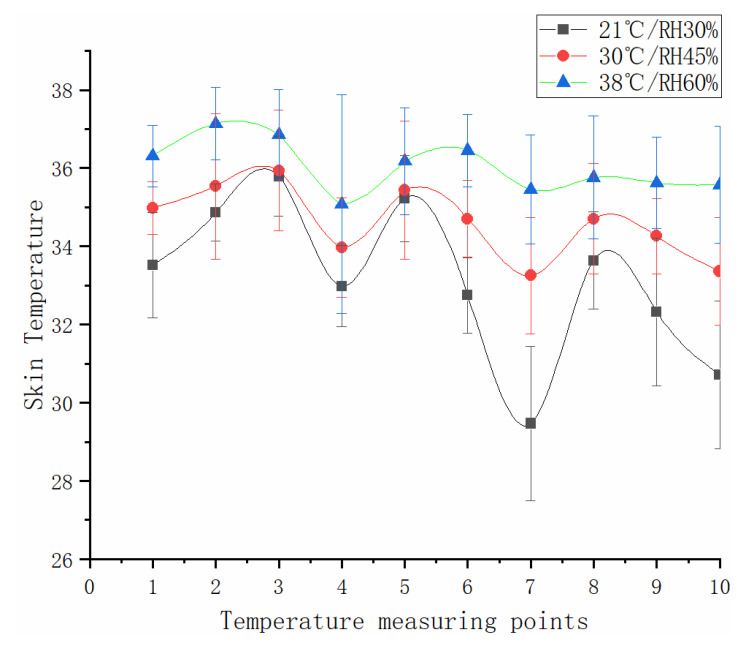
Mean skin temperature changes at various measuring points, where points 1–10 represent of the head, left upper arm, and chest, back, abdomen, left lower arm, left hand, left thigh, left calf, and left foot.

Figure 5 depicts the variations of mean skin temperatures at various body sites during 55 min of simulated flight under 21 °C/30%, 30 °C/45%, and 38 °C/60% RH. As can be seen from Figure 5, the mean skin temperatures of the chest are not much different between 21 °C/30% and 30 °C/45% RH (*p* = 0.850), and the same applied for the abdomen (*p* = 0.889), showing no interaction effect of heat condition and task (p_1_ = 0.968, p_2_ = 0.995), the skin temperature of the other position increased significantly with the increase in ambient temperature (*p* < 0.05).

The skin temperature of the hands was 35.45 °C under 38 °C/60% RH condition, which was 5.99 °C higher than that under 21 °C/30% RH. Compared with the increase in skin temperature in the other points, the change of hand was the biggest (*p* < 0.001), and the interaction of thermal conditions and time on the mean skin temperature of the hands (*p* < 0.01). According to the increased value of each position, the skin temperatures can be classified into three groups: the skin temperature variations of the chest and abdomen increased less than 1 °C, while the skin temperature range of the hands, feet, and forearms was more than 3 °C, and the temperature range of other positions was 1–3 °C. Under a high-temperature environment, there was no difference between the mean skin temperature of each part (*p* > 0.05).

### 3.3. Thermal Sensation

**Figure 6:**
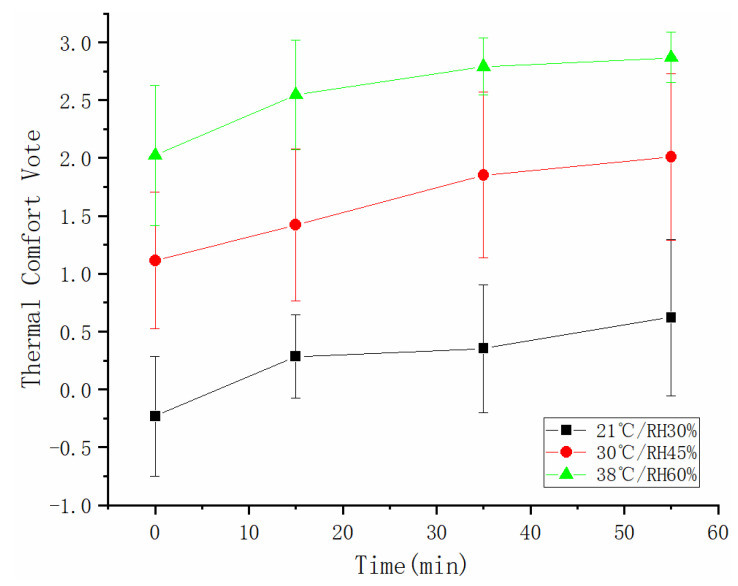
Thermal comfort votes under different environmental conditions.

As shown in Figure 6, there was a difference in thermal comfort vote among three conditions, which increased with rising ambient temperature and humidity (*p* < 0.001). In a 21 °C/30% RH environment, there was no difference in thermal comfort (*p* = 0.077); the subjects were in a relative comfort state, whose votes ranged between 0–0.5, and no interaction effect of heat condition and the task was found (*p* = 0.052). The thermal discomfort increased over time under 30 °C/45% RH (*p* = 0.01) and 38 °C/60% (*p* < 0.001) RH. At 38 °C/60% RH, in particular, the votes ranged between 2–3 with the standard deviation decreased with time, reflecting the extreme discomfort of the subjects.

### 3.4. Sweat Amount

**Figure 7:**
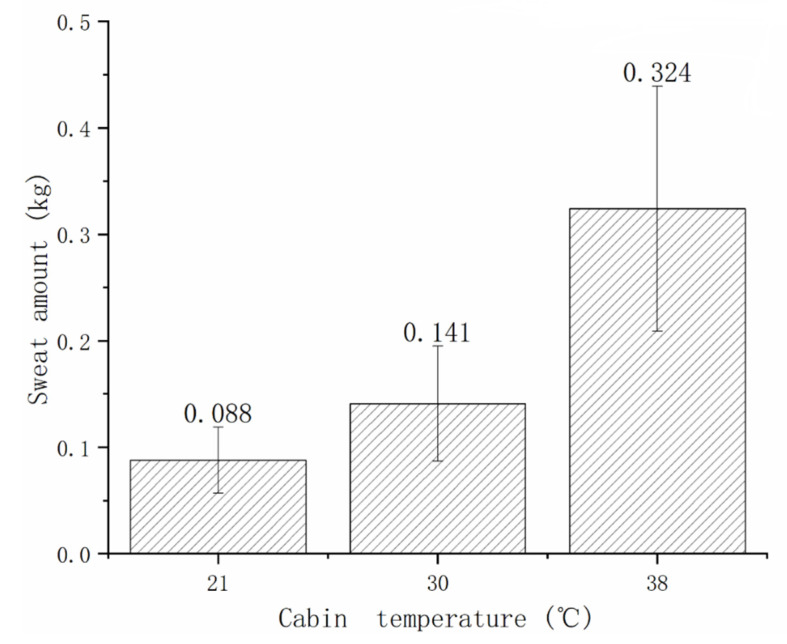
Sweat amounts at various ambient temperatures.

Figure 7 shows that the mean sweat amounts increased significantly with rising ambient temperature under the three experimental conditions. The sweat amounts of subjects at 38 °C/60% RH were higher than those of 21 °C/30% RH and 30 °C/45% RH (*p* < 0.01). However, no difference was observed between conditions at 21 °C/30% and 30 °C/45% RH (*p*= 0.05), as it is known that the body begins to sweat at low wind speeds when the male skin temperature reached 34 °C [28,29,30]. As shown in Figure 6, the sweating was considerably smaller at 21 °C/30% RH, which just reached the critical point, with a mean of 0.088 kg. At 38 °C/60% RH, the sweat amount increased remarkably to 0.324 kg on average. This was equivalent to 0.5% of the mean body weight, which was 3.7 times that at 21 °C/30% RH.

### 3.5. Pulse Rate and SpO2

**Figure 8:**
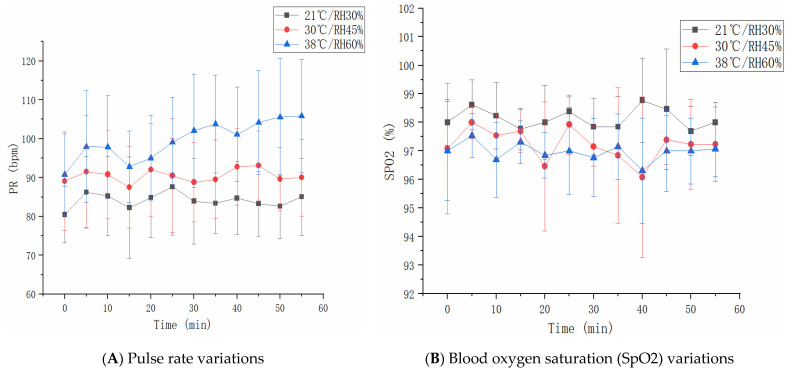
Pulse rate and SpO2 changes under different conditions.

As shown in Figure 8A, there was an interaction effect of heat condition and task on pulse rate (*p* = 0.001), and the pulse rates show inter-condition differences among different tasks (*p* < 0.001). In the 38 °C/60% RH environment, the pulse rate linearly increased over time (*p* < 0.01, r^2^ = 0.78), with a maximum of up to 106 bpm. Under the other two conditions, the temporal changes in pulse rate were not significant. According to the analysis of changes in SpO2 in Figure 8B, there was no interaction effect of heat condition and the task (*p* = 0.746), and no differences were reported between the conditions of 30 °C/45% and 38 °C/60% RH (*p* = 0.146). The level of SpO2 under 21 °C/30% RH was significantly higher than that under the other two conditions (p_1_ = 0.001, p_2_ < 0.001).

### 3.6. Target Strike Performance

**Figure 9:**
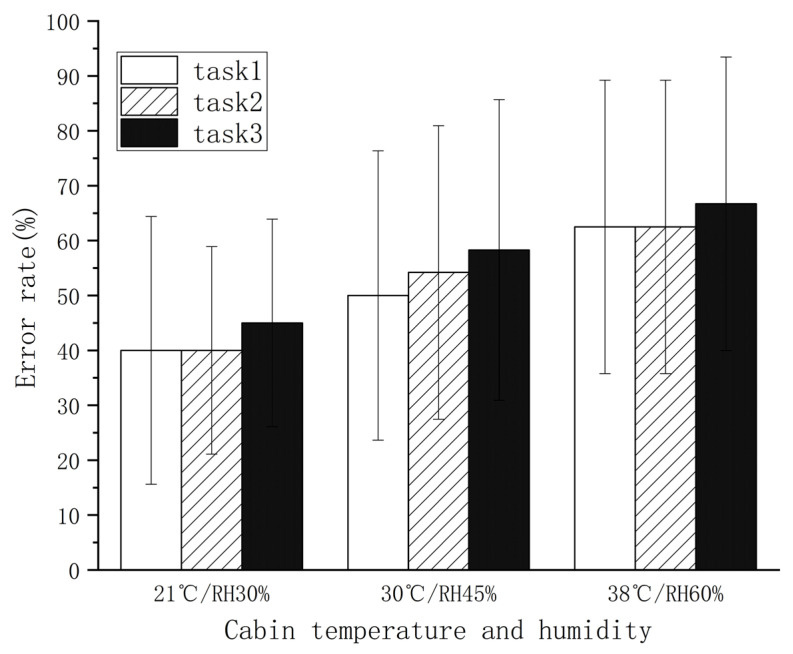
Target strike performances under different experimental conditions.

As shown in Figure 9, the target is operation rates of subjects, not differences during the same missions, which presented slight increases over time (*p* > 0.5). In contrast, with the rise in temperature and humidity, the error rates increased for all three missions (*p* < 0.01). The mean error rate was up to 63.9% in a 38 °C/60% RH environment, which was lower than 60% in a 30 °C/45% RH environment.

To explore major factors affecting the operational performance of pilots, this study analyzed the relationship between operation error rate and pulse rate, skin temperature, and core temperature index by logistic regression analysis. The study found that the experimental data fitted well with the model (r^2^ = 0.81, *p* < 0.05). As shown in Table 2, the core temperature coefficient was 0.146, and the error rate had a positive correlation coefficient with core temperature (*p* = 0.04).

## 4. Discussion

### 4.1. Changes in Body Temperatures under Different Experimental Conditions

According to a study by Guo et al. on changes in the human body’s core temperature during light, moderate, and heavy operations at 32 °C, 36 °C, and 40 °C, the core temperature tended to increase with ambient temperature elevation, which agrees with the finding of this study. In this study, the core temperature reached 38.0 °C after flying and combating for 55 min at 38 °C/60% RH. According to the American Conference Governmental Industrial Hygienists (ACGIH) workload classification, the labor intensity in this paper is also a moderate operation. In Guo’s study, the core temperature was 37.9 °C after 60 min of moderate operation at 36 °C/90% RH [31], which is consistent with this study. According to ISO9886 standards [32], the upper limit of ordinary human core temperature was 38.5 °C. Therefore, subjects should not work more than 55 min in a high temperature and humidity environment without cooling protection. The cause of elevated core temperature was the heightened metabolic rate and energy expenditure of subjects operating in the high temperature and humidity environments, in contrast to the slow heat loss of human skin. Additionally, this study found that the core temperature rose the fastest in the initial 25 min of experimentation under different environments, and then presenting a slow rise over time. The possible reason was that an adaptation and adjustment process was required when people entered into a new environment and eventually adapted to it. The thermoregulation mechanism controls the heat balance of the human body through sweating, convection, and other heat dissipation. In this study, the considerably larger sweat outputs at 38 °C/60% RH than the other conditions were attributed to the difficulty of heat output caused by the partial pressure of saturated vapor on the skin surface from that of water vapor in the air under high temperatures and humidity.

A study by Ye Yao found significant elevations in the mean skin temperature of the human body with the rising ambient temperature (21, 24, 26, and 29 °C), which agrees with the results of the present study [33]. The skin temperature depends on the blood flow and blood temperature. As the ambient temperature heightens, the blood vessels relax, and the blood flow increases to elevate the skin temperature. According to the physiological requirements of pilots, the mean skin temperature can be regarded as the physiological zone and an assessment index of cabin temperature control [34,35,36]. Thermal comfort is guaranteed when the mean skin temperature is 33.0–34.5 °C, whereas a temperature range of 34.6–35.6 °C is regarded as the ergonomic zone [36]. In this study, the mean skin temperatures were 33.5 °C and 34.8 °C, respectively, in the 21 °C/30% and 30 °C/45% RH environments. Clearly, the need for thermal comfort can be met if the cockpit temperature and humidity range between 21 °C/30% and 30 °C/45% RH.

Changes in skin temperature varied by body site. Yao’s study found that the local skin temperatures rose with the elevation of ambient temperature, with the greatest changes observed at the feet, and the fewest changes observed at the chest [33]. The present study also found differences in the skin temperatures at various sites under the same environmental conditions. At high temperatures and humidity, the skin temperature was the highest at the chest and back, whereas the lowest was at the upper arm. With the elevation of ambient temperature and relative humidity, the skin temperatures showed the fewest increases at the back and abdomen, and the greatest increases at the hand and foot. These findings are consistent with Yao’s results. Given the varying skin temperatures over the body, the heat generated was uneven, so rational ventilation quantification was necessary for each local segment of pilot ventilation suits [37]. An exploration of the heat dissipation performance of airtight ventilation suits by Charting et al. found the pipe layout and flow distribution were quite irrational [38]. According to the results of this study, increasing the ventilation flow of the chest and abdomen can improve the effectiveness of thermal comfort.

Additionally, the subjects experienced markedly intensifying thermal discomfort due to the rise in ambient temperature and humidity. Wu et al. found that the thermal sensation of the human body had a linear relationship with the ambient temperature [39]; the intensification of thermal discomfort may be attributed to the increased amount of sweat resulting from high ambient humidity. Brager [40] et al. found three modes of human adaptation through related literature research: behavior, physical, and psychological adaptation. The subjective evaluation results were easily affected by the emotions of the survey subjects. Therefore, in this study, the mean skin temperature, the heart rate, and the blood oxygen were used to predict thermal comfort by Linear Regression. The fitting effect was good (r^2^ = 0.69, *p* < 0.05), and the mean squared error (MSE) was 0.369. This result manifests that these physiological parameters could be effective indicators of human thermal comfort. In addition, we found no significant correlation between the sweating volume of the subjects under these three experimental conditions. In other words, we cannot find out who sweats easily.

The thermal load of the fighter cockpit was mainly related to the pilot’s clothing, labor intensity, electronic equipment, etc. [41,42]. The results of this study could serve as the foundation for individual cooling equipment.

### 4.2. Physiological Responses under Different Experimental Conditions

An upward trend of pulse rate was found with the rising ambient temperature and humidity. Tian et al. studied the temporal changes of human pulse rate during stair walking in the condition of 37.0 °C/40% RH. Consistent with the present finding, the pulse rate tends to increase over time [43]. The increase in pulse rate is attributed primarily to the excitement of cardiac sympathetic nerves upon the rise of the ambient temperature, which stimulated the adrenaline secretion to act on the myocardial cells, thereby resulting in a quickened heart rate. Body temperature also influences pulse rate; the heart rate increases with the increase in core temperature [44,45]. This increase is termed thermal cardiac reactivity (TCR), and also thermal heart rate component [46,47].

SpO2 variation is a vital physiological indicator as well. According to a study by Sun et al., high temperature and humidity environments influence human health by causing markedly increased oxygen consumption, heart failure, and by activating physiological responses such as hypoxia [48]. This study also discovered a drop in SpO2 with the rising temperature and humidity, which is in agreement with Sun’s result. The probable cause of the declining SpO2 is the decrease in blood hemoglobin levels at invariant ambient pressure and the increased ambient temperature.

### 4.3. Operational Performances under Different Experimental Conditions

In the high-temperature environment, the minimum perception and reaction flexibility of fighter pilots decrease [49]. An upward trend in the operational error rate of subjects was found with the rising ambient temperature and humidity, which is consistent with Tian’s research. To figure out the reasons for this phenomenon, the correlations between the increase in the operational error rate and the changes in physiological indicators were analyzed. The error rate was found to be significantly correlated with the core temperature. According to Reinertsen and FÆREVIK’s study, the elevation of core temperature was because of reduced vigilance and increased operational errors among pilots [21]. Allan et al. analyzed the impact of the increase in ear canal temperature on flight crew efficiency. They found that when the ear canal temperature reached 37.9 °C, the ability to track moving targets decreased by 13.6% [50]. Additionally, the presence of certain errors in 21 °C/30% RH was attributed to the inadequate operational proficiency and expertise of the subjects, who were not professionally trained pilots. Nevertheless, these errors did not affect the present results since the research focus was relative error rates under various conditions. In addition, there are numerous sensory nerves in the skin, whose sensitivity can be affected by a high-temperature. This consequently leads to slower nerve conduction or decreased accuracy, thereby resulting in discomfort and cognitive decline. Research by Gillingham suggested that the operational performance of pilots could be affected significantly by 1.2% dehydration [51]. In this study, the maximum dehydration rate was 0.5%, which was not related to the heightened error rate. Therefore, the rise in human body temperature is a critical cause of operation errors.

Bart and Rasmussen’s research found that the hot and humid environment caused changes in the body’s related neurobehavioral capabilities by changing the content of neurotransmitters [52,53]. The heat condition influenced signal transduction pathways and stimulated immune responses [54], further aggravating the impact on the pilot’s neurobehavioral ability. Under the high-temperature conditions, the response time of subjects is significantly prolonged, and the correct response rate is reduced [55,56], and these conclusions are consistent with the present result.

The present study is limited by its single sample source. Only volunteer college students were taken as samples, who somewhat differed in the physical constitution and operational skills of the professional pilots. In addition, further research is needed concerning the physiological characteristics and combat performance of female pilots in high-temperature environments. In future research, we will add more temperature measurement methods, such as an oral cavity or intestinal tract.

## 5. Conclusions

In this study, the physiological characteristics of pilots are explored during simulated combat and under more real fighter cockpit environments. Under high temperature and humidity combat conditions, the core temperatures are at fever levels, which produces an adverse impact on human physiological functions. The probability of flight errors increases significantly during combat under high temperature and humidity conditions. In addition, the core temperature was related to the operational mean error rate. This study found that thermal comfort could be predicted by core temperature, skin temperature, and heart rate; according to the mean skin temperature range for thermal comfort, the temperature and RH of aircraft cabin environment is recommended to be controlled between 21 °C/30% and 30 °C/45% RH. Concerning the design of ventilation suits, it is recommended to increase the air flow or the ventilation diameter of the pilot’s chest and abdomen.

## Figures and Tables

**Table 1:** ASHRAE seven-point thermal sensation scale.

ASHRAE Thermal Sensation	
+3	Hot
+2	Warm
+1	Slightly warm
0	Neutral
−1	Slightly cool
−2	Cool
−3	Cold

**Table 2:** The logistic regression parameters of operation error rate vs. core temperature.

	Coefficients	t	*p*-Value
Intercept	−4.58	−3.01	0.02
Pulse Rate	0.0015	0.32	0.76
Skin Temperature	−0.01	−0.44	0.68
Core Temperature	0.146	2.6	0.04

## Data Availability

Not applicable.
